# Scalable Production of Low-Molecular-Weight Chitosan: Comparative Study of Conventional, Microwave, and Autoclave-Assisted Methods

**DOI:** 10.3390/polym18020213

**Published:** 2026-01-13

**Authors:** Mithat Çelebi, Abdullah Tav, Mehmet Arif Kaya, Zafer Ömer Özdemir

**Affiliations:** 1 Department of Polymer Materials Engineering, Faculty of Engineering, Yalova University, Yalova 77200, Türkiye; 2 Polymer Materials Engineering, Institute of Graduate Studies, Yalova University, Yalova 77200, Türkiye; 3 Hamidiye Pharmacy Faculty, University of Health Sciences, İstanbul 34668, Türkiye

**Keywords:** chitin, chitosan, deacetylation, FT-IR, autoclave-assisted method, microwave energy, thermochemical method, molecular weight, green chemistry

## Abstract

The valorization of shrimp shell waste is crucial for promoting sustainability and a circular economy. This study aimed to extract chitin from the exoskeletal residues of deep-water rose shrimp (*Parapenaeus longirostris*) sourced from the Marmara Sea and synthesize low-molecular-weight chitosan (LMWC) via conventional, microwave-, and autoclave-assisted deacetylation pathways. The shell biomass was subjected to sequential demineralization (1 M HCl) and deproteinization (1 M NaOH), yielding 14.42% chitin. The extracted chitin was then converted to LMWC using the three methods, and the products were characterized using FT-IR spectroscopy, titration, viscometry, SEM, and TGA. The results demonstrated that the autoclave-assisted method achieved the highest degree of deacetylation (DD) at 95%, significantly outperforming the conventional method (81%) and the microwave-assisted method (67%). The autoclave-synthesized chitosan also exhibited the lowest viscosity (33 cP), confirming its low molecular weight. Morphological analysis showed that chitin exhibited a well-defined fibrous structure. After deacetylation, this structure transformed into a rough and porous surface morphology. Thermal analysis further demonstrated that the laboratory-synthesized chitosan exhibited higher thermal stability than the commercial chitosan sample. In conclusion, the autoclave-assisted method proved to be highly efficient for producing low-molecular-weight chitosan with a high degree of deacetylation. However, the conventional method remains the most practical option for scalable industrial production due to its simplicity and well-established infrastructure. Moreover, the laboratory-synthesized chitosan exhibited higher thermal stability, increased porosity, and a higher degree of deacetylation compared to commercially available chitosan, which may offer functional advantages in applications requiring enhanced reactivity, solubility, or thermal resistance. Overall, the findings provide valuable insights into selecting appropriate deacetylation strategies for producing low-molecular-weight chitosan with tailored properties, thereby bridging the gap between laboratory-scale synthesis and potential industrial applications.

## 1. Introduction

Crustacean shell valorization has gained increasing attention due to its alignment with global sustainability and circular economy strategies [1]. The 2030 Agenda for Sustainable Development emphasizes the significance of fisheries and seafood products in promoting international food security and nutrition. Global shrimp production remains one of the dominant segments of crustacean fisheries and aquaculture. Capture fisheries accounted for approximately 3.3 million tons of shrimp production in 2022, while aquaculture production, dominated by penaeid shrimp species, reached about 7.9 million tons, representing nearly 62% of total crustacean aquaculture output [2,3]. In Türkiye, crustacean production reached approximately 2471 tons in 2024, predominantly consisting of *Parapenaeus longirostris* (deep-water rose shrimp), which is commonly harvested from the Marmara Sea [4]. Despite their economic importance, shrimp processing generates substantial amounts of waste, with up to 60% of the total biomass discarded as shells, heads, and tails. These byproducts are highly perishable and may pose environmental and public health concerns due to microbial degradation and unpleasant odors, leading to their underutilization [5]. Nevertheless, shrimp shell waste is rich in chitin, a valuable biopolymer that serves as a key precursor for chitosan synthesis, offering significant potential for sustainable and value-added bioproduct development [6].

Chitin is a linear polysaccharide composed of β-(1→4)-linked N-acetylglucosamine units. Due to its high crystalline and hydrophobic nature, chitin exhibits poor solubility and low chemical reactivity, which severely limits its direct industrial applications [7]. Extensive intra- and intermolecular hydrogen bonding, particularly in the α-chitin form commonly found in the exoskeletons of crustaceans such as shrimp and crabs, contributes significantly to its highly crystalline structure. This high crystallinity, together with the polymer’s hydrophobic nature, results in dense molecular packing, rendering chitin insoluble in most solvents and chemically inert under mild processing conditions [8]. Chitosan is a partially deacetylated derivative of chitin produced through alkaline hydrolysis. It is biodegradable, biocompatible, and exhibits antibacterial activity. Due to these features, chitosan is used in multiple areas including food, agriculture, wastewater treatment, medicine, and pharmaceuticals [9,10]. The production method has a significant influence on the physicochemical properties of the resulting chitosan. This process consists of three main steps: demineralization (DM), deproteinization (DP), and deacetylation (DA) [11].

DM uses acids, often hydrochloric acid (HCl), to remove mineral content, whereas DP uses alkaline solutions, most frequently sodium hydroxide (NaOH), to remove proteins. These two processes produce chitin, which is then transformed into chitosan via DA in concentrated NaOH solutions at high temperatures for extended periods of time. Conventional extraction techniques are energy-intensive, time-consuming, and environmentally hazardous. Innovative methods, such as microwave and autoclave-assisted approaches, have been developed to enhance the efficiency and sustainability of chitosan manufacturing. Microwave-assisted heating has emerged as a potential alternative to conventional heating, as it dramatically shortens extraction time, reduces energy consumption, and provides a more efficient and environmentally friendly approach to producing chitosan [11].

The molar fraction of deacetylated glucosamine units in the polymer chain, known as the degree of deacetylation (DD), is a crucial parameter that governs the physicochemical properties and application potential of chitosan. DD significantly affects solubility, viscosity, and biological activity. Generally, a DD above 70% is required to achieve adequate solubility and bioactivity [12,13]. Additionally, low-molecular-weight chitosan (LMWC) is particularly desirable for applications such as drug delivery and food coatings due to its improved solubility and bioavailability [10].

Microwave-assisted processing is based on the interaction between microwave radiation and polar molecules, resulting in rapid and homogeneous volumetric heating [14]. This mechanism substantially shortens reaction time and reduces energy consumption by accelerating deacetylation, enhancing alkali diffusion, and promoting structural disruption of the polymer matrix. Owing to these advantages, microwave irradiation has been increasingly applied in chemical processing, offering faster reaction rates, higher yields, and improved energy efficiency compared to conventional heating methods [15,16]. Similarly, autoclave-assisted techniques have gained attention as effective alternatives to produce chitosan. By employing saturated steam at 121 °C and 15 psi, autoclave processing combines thermal and pressure effects to improve deacetylation efficiency and facilitate alkali penetration into highly crystalline regions of chitin [17,18]. Overall, both microwave and autoclave-assisted methods represent greener, more energy-efficient alternatives to conventional processing, offering significant potential for scalable industrial chitosan production.

In addition to comparing conventional, microwave-assisted, and autoclave-assisted methods, this study highlights the potential for scalable production of LMWC from crustacean shell waste. Although alternative techniques can significantly reduce reaction time and improve process efficiency, conventional methods continue to offer clear advantages in terms of operational reliability, process simplicity, and compatibility with existing industrial infrastructure [11,19,20,21]. The well-established nature of conventional protocols ensures consistent product quality, lower initial investment costs, and fewer technical challenges during scale-up [22].

Furthermore, waste valorization through conventional extraction remains a practical and economically viable pathway for converting crustacean residues into value-added biopolymers. The scalable production of LMWC also supports the circular economy principles, enabling the transformation of crustacean waste into high-performance biopolymers suitable for applications in pharmaceuticals, food packaging, agriculture, and wastewater treatment [23,24]. While microwave irradiation and autoclave-assisted processes represent promising alternatives for greener and time efficient production, the conventional approach—particularly when optimized—provides a robust, reproducible, and cost-effective strategy for large-scale chitosan manufacturing. Overall, this study demonstrates that, despite the expanding opportunities offered by emerging technologies, conventional extraction remains the most advantageous option when reliability, economic feasibility, and industrial applicability are considered [19,25].

This study aims to systematically compare conventional, microwave-assisted, and autoclave-assisted deacetylation methods to produce low-molecular-weight chitosan from *Parapenaeus longirostris* shell waste. The novelty of this work lies in the direct and controlled comparison of these three deacetylation methods using the same raw material, combined with a comprehensive evaluation of the degree of deacetylation, viscosity, morphology, and thermal stability, benchmarked against commercial chitosan. Through this approach, the study demonstrates to clarify process–structure–property relationships and to identify the most suitable production strategy in terms of material performance and industrial scalability.

## 2. Materials and Methods

Hydrochloric acid (ACS reagent, 37%) and sodium hydroxide (reagent grade, ≥98%) were purchased from Merck (Darmstadt, Germany). Acetone (reagent grade, ≥98%), sodium hypochlorite (NaClO), ethyl alcohol (purity ≥ 96%), and hydrogen peroxide (H_2_O_2_) were obtained from Bereket Kimya (İstanbul, Türkiye). Commercial low-molecular-weight chitosan was purchased from Sigma-Aldrich (St. Louis, MO, USA) (Lot No: 448869). A Nuve OT 012 brand (15 psi pressure capacity) autoclave was used, and a Vestel MD 20 MB 20-L model microwave oven was employed.

Deep-water rose shrimp *Parapenaeus longirostris* was provided with shell waste by a local fish restaurant in Yalova province, located on the coast of the Marmara Sea in Türkiye.

### 2.1. Extraction of Chitin

The shrimp shell waste (SSW) was first washed thoroughly to remove residual impurities and then sun-dried at the coast of Yalova University campus. The dried material was ground using a Renas brand herb grinder operating at 28,000 rpm for 2 min. Moisture content was determined using a gravimetric method [26]. The ground waste was stored in a freezer at −18 °C until use. Demineralization was performed for 1, 2, 4, 6, or 8 h at room temperature using 1.0 N HCl at a solid-to-solvent ratio of 1:15 (*w*/*v*). The demineralized shell was washed with distilled water until the pH reached neutrality, and then dried in an oven at 60 °C. The remaining mineral content after demineralization (DM) was quantified gravimetrically by ash analysis [27]. The mineral content (MC) of SSW was calculated using Equation (1).

(1) $$\mathrm{MC}(\%)=\frac{\left(\mathrm{Weight}\;\mathrm{Loss}\;\mathrm{after}\;\mathrm{DM}-\mathrm{Ash}\;\mathrm{Weight}\;\mathrm{in}\;\mathrm{DemineralizedShell}\right)}{\mathrm{Initial}\;\mathrm{Weight}\;\mathrm{Shell}}\;\times \;100$$

To remove proteins, demineralized shells were treated with 1.0 N NaOH at 80 °C for 4 h at a concentration of 1:15 (*w*/*v*). The washing and drying procedures with distilled water were repeated, as described above. The residual protein content of chitin was determined using a Shimadzu UV-1800 ultraviolet–visible (UV-Vis) spectrophotometer, following a method adapted from the literature [21]. Briefly, 1 g of chitin was treated with 1 M NaOH for 4 h and then filtered. The UV-Vis absorbance of the filtrate was measured at 260 and 280 nm, and the protein content of chitin was determined using Equation (2).

(2) $${\left[\mathrm{Protein}\right]}_{\frac{\mathrm{mg}}{\mathrm{mL}}}=\left[\left(\mathrm{Absorbanc}e_{280}\;\times \;1.31\right)-\left(\mathrm{Absorbanc}e_{260}\;\times \;0.57\right)\right]$$

### 2.2. Decolorization

Decolorization was performed using four different agents (hydrogen peroxide, acetone, ethyl alcohol, and sodium hypochlorite) under sunlight for 4 h. A 3% (*v*/*v*) hydrogen peroxide solution was employed, following a modified version of Tokatlı and Demirdöven’s study [28]. The bleaching process included suspending the material in an 80% (*w*/*w*) ethanol-water and acetone-water solution [29]. The chitin was bleached using a 10% (*v*/*v*) sodium hypochlorite solution. The solid–liquid ratio used was 1:10 (*w*/*v*) for the decolorization experiments. The decolorized chitin samples were dried overnight in an oven at 60 °C [30].

### 2.3. Deacetylation

Deacetylation was performed to synthesize chitosan from the extracted chitin. The chitin was treated with a 50% NaOH (*w*/*v*) solution using different methods at a solid-to-solvent ratio of 1:15 (*w*/*v*). LMWCs were produced using conventional, autoclaved-assisted, and microwave energy-assisted techniques. The conventional method employs two temperatures regimes: 120 °C (the traditional method) and 70 °C (the low-temperature method). The deacetylation conditions and sample codes of the LMWCs produced from chitin extracted using the different approaches are summarized in Table 1.

### 2.4. Degree of Deacetylation

The degree of deacetylation (DD) of chitosan was determined using Fourier-transform infrared spectroscopy (FT-IR) and a titration method. FT-IR spectra were recorded by scanning 16 times at a resolution of 4 cm^−1^ over the range of 4000–650 cm^−1^ using a Perkin Elmer Spectrum 100 FT-IR device equipped with an ATR accessory. The DD of chitosan indicates the number of acetyl groups in chitin that are converted to free amine groups [31]. Equation 3 was used to assess the degree of deacetylation using FT-IR spectroscopy, employing the absorbance 3450 cm^−1^ and 1655 cm^−1^ [32]

(3) $$\mathrm{DD}\left(\%\right)=100-\left(\frac{A1655}{A3450}\times \frac{100}{1.33}\right)$$

**Figure 1:**
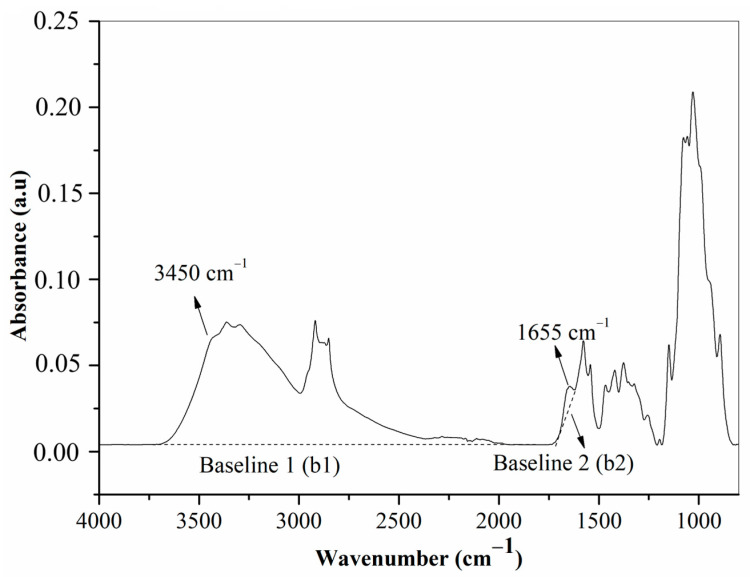
Baselines and reference bands in FT-IR spectra to calculate the degree of deacetylation of chitosan.

Figure 1 illustrates baseline 1 (b1) and baseline 2 (b2), which were used to calculate the degree of deacetylation (DD) of chitosan in the FT-IR spectra. The DD was calculated using Equation (3) based on the absorbance at 3450 cm^−1^ (–OH stretching) and 1655 cm^−1^ (amide I band). The degree of deacetylation of chitosan was calculated using Equation (4), based on the titration method described in a previous study. Chitosan (0.200 g) was dissolved in 20 mL of 0.1 N HCl solution. Distilled water (50 mL) was added to the solution. The resulting solution was titrated with 0.1 N NaOH [32].

(4) $$\mathrm{DD}\left(\%\right)=2.03\;\times \;\frac{V_{2}-V_{1}}{m+0.0042\;\times \;\left(V_{2}-V_{1}\right)}\;$$

where m, V_1_, and V_2_ are the weight of the sample and volumes of 0.1 N NaOH solution corresponding to the equivalence points of the titration curve, respectively.

### 2.5. Solubility and Viscosity Determination

The solubility of the obtained chitosan samples was determined in 1% (*v*/*v*) acetic acid solution following a gravimetric method. Briefly, 0.500 g of dried chitosan was dispersed in 50 mL of 1% (*v*/*v*) acetic acid solution to obtain a 1% (*w*/*v*) chitosan suspension. The mixture was stirred at 250 rpm and 25 °C for 8 h to ensure sufficient interaction between the polymer and solvent. After stirring, the suspension was filtered under vacuum using quantitative filter paper to remove any undissolved fraction. The clear filtrate, containing the dissolved chitosan, was collected and frozen at −18 °C for 12 h, followed by freeze-drying (lyophilization) to recover the soluble chitosan fraction. The solubility (%) of chitosan was calculated gravimetrically by comparing the initial dry mass of chitosan with the dry mass of chitosan recovered after lyophilization, according to the following Equation (5):

(5) $$\mathrm{Solubility}\;(\%)=\left(\frac{m_{\mathrm{dissolved}}}{m_{\mathrm{initial}}}\right)\times 100$$

where m_initial_ was the initial dry mass of chitosan (0.500 g) and m_dissolved_ was the dry mass of the dissolved chitosan obtained after freeze-drying.

The viscosity of the chitosan solutions was measured using a Brookfield DV2 viscometer at 150 rpm and 25 °C using Spindle No. 2. The 1% (*w*/*v*) chitosan solutions were dissolved in 1% acetic acid at 25 °C, and the solution viscosity was given in centipoise (cP).

### 2.6. SEM Analysis

The samples were coated with a thin layer of gold to acquire topographic images, and their surface morphologies were analyzed using FEI Inc. (Hillsboro, OR, USA) Inspect S50 scanning electron microscope (SEM) at an acceleration voltage of 7.5 kV. SEM micrographs were obtained at 200×, 10,000×, 10,000×, and 50,000× magnifications. Elemental analysis of pink shrimp shell waste was performed using an Octane Prime Model energy-dispersive spectroscopy (EDS) device.

### 2.7. TGA

Thermogravimetric analysis (TGA) was conducted using a Seiko Exstar S II 6300 TG/DTA thermal analyzer (Seiko Instruments Inc., Chiba, Japan) in a nitrogen atmosphere. The thermal behavior of the samples was analyzed at a heating rate of 10 °C/min over a temperature range of 20–700 °C [33].

### 2.8. Ash Analysis

The ash content of the chitin samples was calculated using the gravimetric method [27]. First, the samples (5.0 g) were burned, resulting in a completely coal-like and tarry appearance. The sample was then incinerated in a muffle furnace at 600 °C for 4 h. The residue was allowed to cool to 200 °C and quickly transferred to a vacuum desiccator, to prevent exposure to air. Finally, the samples were allowed to return to room temperature. The results were evaluated by weighing the crucible with the ash residue against the initial sample weight.

## 3. Results

### 3.1. Pretreatment, Drying, and Grinding

**Figure 2:**
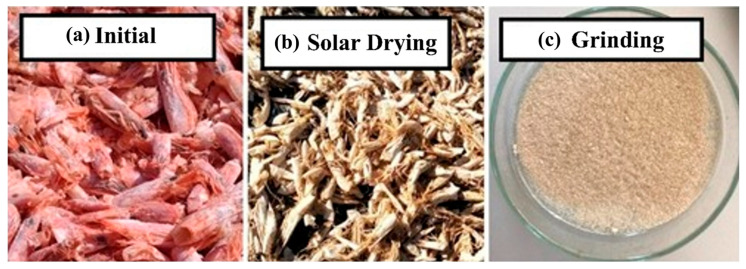
Digital images of (**a**) shrimp shell waste; (**b**) shrimp shell waste after solar drying; (**c**) grounding of solar drying shrimp shell waste.

Pretreatment steps, including washing, sun-drying, and grinding, enhanced the efficient chemical extraction. Plant residues and other impurities were removed from shrimp shell waste, which were subsequently rinsed with distilled water and sun-dried. The water content of the shrimp shell waste was 73.2 ± 5.4%. Figure 2 shows three different forms of shrimp shell waste: (a) initial, (b) sun-dried, and (c) ground. After drying, the shrimp shell waste was ground using a high-speed grinder (28,000 rpm for 2 min).

### 3.2. Chitin Extraction Performance

#### 3.2.1. Demineralization

Table 2 summarizes the effect of acid treatment time on the demineralization of shrimp shell waste (SSW). An initial comparison using 25 g of ground and unground shrimp shell waste treated with 1 N HCl for 4 h revealed that ground shells achieved higher demineralization efficiency (39.76% vs. 51.12% ash residue). Therefore, all subsequent experiments were conducted using ground shrimp shell waste. Subsequently, 200 g of shrimp shell waste were treated with 1 M HCl at room temperature for 1, 2, 4, 6, and 8 h. The amount of minerals remaining after demineralization was evaluated using ash analysis. As shown in Table 2, the ash residue of shrimp shell waste decreased significantly, 2.42% and 0.9% after 2 and 8 h, respectively.

The mineral percentage of the SSW was calculated as 54.31 ± 2.29%, based on the average of the mineral contents from the five experiments, shown in Table 2.

#### 3.2.2. Deproteinization

Table 3 presents the proximate chemical composition of pink shrimp shell (*Parapenaeus longirostris*) waste collected from the Marmara Sea, including its moisture, mineral, protein, and chitin fractions, as well as the elemental mineral distribution (Ca, P, Mg) obtained by SEM–EDS. Initially, 20 g of demineralized SSW were treated with 1 M NaOH solution at 80 °C for 1, 2, and 4 h, yielding 7.61 g, 8.1 g, and 8.03 g of chitin, respectively, after washing and drying. After confirming that 4 h was sufficient for protein removal, the deproteinization process was conducted on 200 g of demineralized shrimp shell waste. At the end of the 4 h treatment, 64.66 g of product remained. The residual protein content in this product was determined to be 2.4 ± 4.41%. Based on the demineralization results, the dried shrimp shell waste was calculated to contain 54.31%, 31.27%, and 14.42% of mineral, protein, and chitin, respectively.

#### 3.2.3. Visual Comparison of Decolorization Treatments

**Figure 3:**
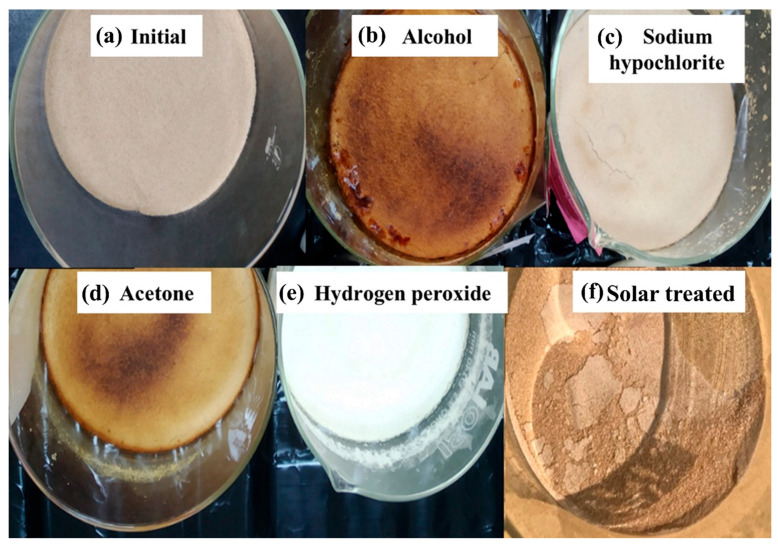
Digital images of chitin samples (**a**) initial (non-treated), (**b**) alcohol, (**c**) sodium hypochlorite, (**d**) acetone, (**e**) hydrogen peroxide, (**f**) solar-treated samples.

As shown in Figure 3, the effects of different decolorization methods, including treatment with sodium hypochlorite, acetone, ethanol, hydrogen peroxide, and solar drying, on chitin samples were evaluated. Both chemical agents and solar energy were assessed for their ability to remove the pigments. As shown in Figure 3, although significant bleaching occurred with hydrogen peroxide, treatment with ethanol (b) and acetone (d) resulted in browning after drying. Sodium hypochlorite (c) yielded moderate results, whereas sun treatment (f) only whitened the chitin surface. Chitin is closely associated with proteins, lipids, and pigments in its natural biological matrix [34]. Among these pigments, astaxanthin is the dominant one, and when it is not entirely removed during purification, it may undergo oxidation reactions. As a result, astaxanthin degradation products form new chromophoric structures, leading to the brown/dark coloration observed in Figure 3b,d. Therefore, the brown appearance is mainly attributed to the oxidative transformation of residual astaxanthin and other organic impurities [35,36]. In contrast, oxidative bleaching agents such as sodium hypochlorite (Figure 3c) and hydrogen peroxide (Figure 3e) these pigments, resulting in significantly brighter samples.

### 3.3. Deacetylation Methods and Degree of Deacetylation

Deacetylation of chitin by microwave energy was carried out with 50% NaOH (*w*/*w*) at 140 W in 15 min, and a deacetylation degree of 67% was achieved. As a result of the deacetylation process performed in an autoclave at 121 °C for 1 h, the chitin extracted from the shells yielded approximately 11%. In contrast, the thermochemical method at 121 °C for 4 h produced 8% chitosan. Chitosan was produced with an 81% yield by chitin deacetylation using the conventional method. FT-IR spectroscopy and titration were used to determine the degree of deacetylation of chitosan produced by various methods. The deacetylation values of chitosan produced by deacetylation of chitin using various methods and conditions in recent years, along with the results obtained in this study, are presented in Table 4.

Furthermore, this study found that the deacetylation degree of chitosan synthesized with 50% NaOH was 83% at 120 °C and 180 min. It was characterized as a low-molecular-weight chitosan with a viscosity ranging from 20 to 300 cPs. Table 4 shows that the viscosity of the chitosan synthesized in this study ranged from 33 to 117 cP. Viscosity measurements confirmed a decrease in the molecular weight with increasing temperature and reaction time. Although viscosity is affected by both molecular weight and DD, viscometry does not provide an absolute method for determining molecular weight or polydispersity. As highlighted in previous studies, molecular weight distribution requires chromatographic or scattering techniques for analysis. Therefore, the viscosity data presented were interpreted as indirect indicators of molecular characteristics [37]. According to the literature, solubility in 1% acetic acid was found to correlate with the degree of deacetylation. The chitosan samples with DD ≥ 81% were completely soluble, though the chitosan with DD ~64% showed partial dissolution behavior with insoluble residues [13].

**Table 4:** The deacetylation conditions of the produced chitosan from extracted chitin by using different methods.

Raw Material	Method	NaOH (%)	Temp. (°C)	Reaction Time	Yield (%)	FT-IR (DD)	Titration (DD%)	Solubility (%)	Viscosity (cPs)/Molecular Weight (kDa)	References
Shrimp shell *Parapenaeus longirostris*	Conventional	50	120	3 h.	76	81	77	95.1	117 cPs	This Study
	Low Temperature	50	70	48 h.	*N.D	79	71	91.3	*N.D	This Study
	Microwave energy 140 W	50		15 min.	*N.D	67	64	77.5	55	This Study
	Autoclave-assisted	50	121	60 min.	*N.D	83	95	97.6	33 cPs	This Study
	Commercial	*N.D	*N.D	*N.D	*N.D	72	85	95.5	102 cPs	This study
Shrim shell (*Litopenaeus vannamei)*	Autoclave-assisted	80	91.2	45 min.	85	83.3	*N.D	*N.D	*N.D	[17]
Shrim shell *(Metapenaeus dobsoni)*	freeze-pump-out-thaw cycle	12.5 M, frozen, 24 h.	90	240 min.	*N.D	97.2	*N.D	98.1	620 kDa	[13]
White Pacific shrimp shell waste (*Litopenaeus vannamei*)	Chemical Method (%10 HCl)	50	50	120	15	93.8	*N.D	*N.D	*N.D	[38]
Chitin from Crustacean	Autoclave- assisted	50	121	30	50	80	*N.D	90	123 cP	[39]
Commercial chitin	Autoclave- assisted	50	121	30		92	*N.D	97.4	2230 cP	[18]
Shrimp shell (*Litopenaeus vannamei)*	Microwave energy 600 W	45		15	43	81	*N.D	*N.D	*N.D	[40]
Shrimp shell Taiwan	Conventional	40	100	12 h	*N.D	93	*N.D	*N.D	*N.D	[41]
Shrimp waste	Conventional	40	120	300 min.	77	66	78	*N.D	120 cPs	[28]
Shrimp shell	Conventional	50	100	720 min.	74	80	85	*N.D	40 cPs	[28]
Shrimp shell (*Parapenaeus longirostris)*	Microwave energy 90, 160, 350, 500, 650 W	50		14 min.	*N.D	51, 57, 75, 80, 85	*N.D	*N.D	*N.D	[21]
Commercial chitin	Microwave energy 900 W	45		5.5 min.	*N.D	85	*N.D	*N.D	*N.D	[42]

*N.D: no data.

### 3.4. Characterization

#### 3.4.1. Morphological Analysis

**Figure 4:**
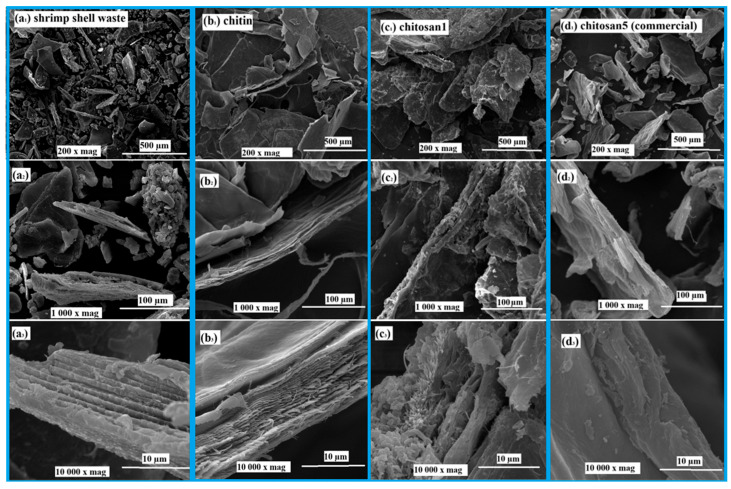
SEM micrographs of (**a1**). shrimp shell waste at 200× magnification, (**a2**). shrimp shell waste at 1000× magnification, (**a3**). shrimp shell waste at 10,000× magnification, (**b1**) chitin at 200× magnification, (**b2**). chitin at 1000× magnification, (**b3**). chitin at 10,000× magnification, (**c1**). chitosan1 at 200× magnification, (**c2**). chitosan1 at 1000× magnification, (**c3**). chitosan1 at 10,000× magnification, (**d1**). chitosan5 at 200× magnification, (**d2**). chitosan5 at 1000× magnification, (**d3**). chitosan5 at 10,000× magnification.

Figure 4 presents SEM micrographs of shrimp shell waste (a), chitin (b), laboratory-produced chitosan1 (c), and commercial chitosan5 (d) at three magnifications: 200×, 1000×, and 10,000×. At 200× magnification, shrimp shell waste (Figure 4(a1)) displayed uneven, compact, and mineral-rich segments, whereas chitin (Figure 4(b1)) exhibited a more structured fibrous morphology after demineralization and deproteinization. Laboratory-synthesized chitosan (Figure 4(c1)) exhibited a rougher and more irregular surface than chitin. Commercial chitosan (Figure 4(d1)) appeared smoother and more homogeneous. At 1000× magnification, shrimp shell waste (Figure 4(a2)) retained residual mineral deposits across the fibrous zones, whereas chitin (Figure 4(b2)) showed continuous, well-defined fibrous layers. Chitosan1 (Figure 4(c2)) demonstrated partial disruption of this fibrous alignment, leading to irregular morphologies, whereas commercial chitosan (Figure 4(d2)) showed a denser and more consistent surface. At 10,000× magnification, shrimp shell waste (Figure 4(a3)) exhibits crystallized and compact domains, whereas chitin (Figure 4(b3)) exhibits highly aligned fibrillar layers. In contrast, Chitosan1 (Figure 4(c3)) exhibited a fragmented and heterogeneous surface. Commercial chitosan (Figure 4(d3)) appeared to be more compact and smoother, although small microaggregates were visible on its surface.

**Figure 5:**
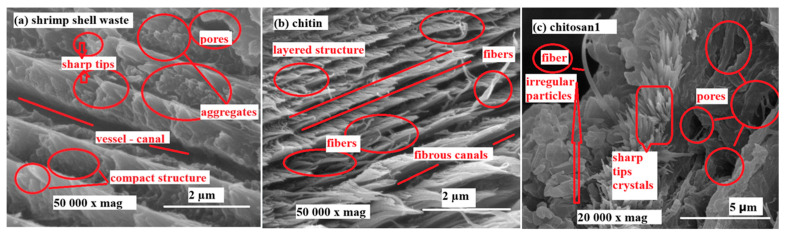
SEM micrographs of (**a**) shrimp shell waste 50,000× mag, (**b**) chitin 50,000× mag, (**c**) chitosan1 20,000× mag.

Figure 5 presents SEM micrographs of shrimp shell waste (a), chitin (b), and laboratory-produced chitosan1 (c), captured at high magnification (50,000× for shrimp shell waste and chitin, 20,000× for chitosan1). Figure 5a highlights the compact and dense morphology of shrimp shell waste, characterized by claw-like projections, sharp tips, and mineral–protein aggregates. As shown in Figure 5b, the fibrous architecture of chitin is clearly visible after the removal of minerals and proteins. Parallel layered structures were apparent on the surface, and microchannels were linked to these layers. Figure 5c presents the SEM micrograph of chitosan1 at 20,000× magnification, where partial degradation of the fibrous chitin network is evident. The surface appears rougher and more porous.

#### 3.4.2. FT-IR Spectra of Extracted Chitin

**Figure 6:**
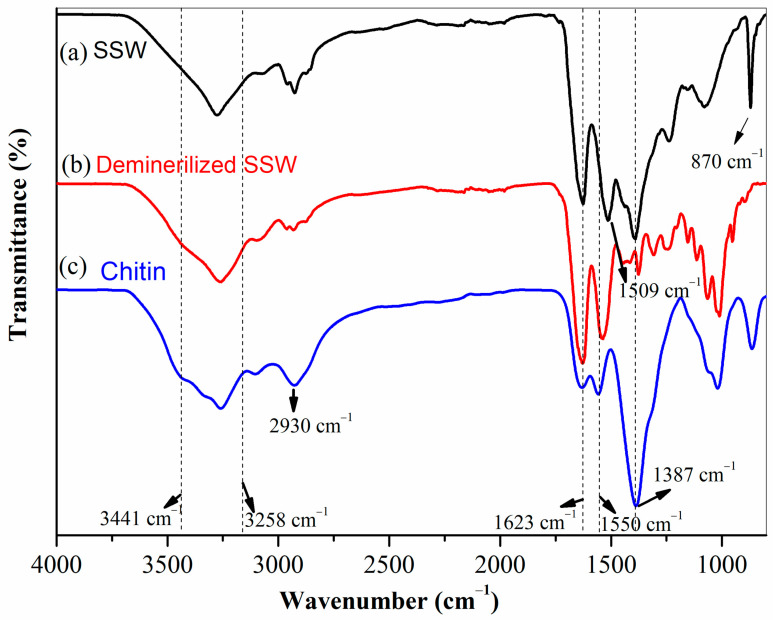
FT-IR spectrum of (**a**) shrimp shell waste; (**b**) demineralized shell; (**c**) chitin.

Shrimp shells are composed of chitin, proteins, minerals (primarily calcium carbonate), lipids, and pigments, which can be identified by FT-IR analysis [43]. The FT-IR spectra of (a) shrimp shell waste, (b) demineralized shrimp shell waste (after 4 h of demineralization), and (c) chitin obtained after 4 h of deproteinization are shown in Figure 6. The broad bands between 3441 cm^−1^ and 3258 cm^−1^ are attributed to the O–H and N–H stretching vibrations of the hydroxyl and amine groups, respectively, which are present in all samples. In Figure 6a, the band at 1623 cm^−1^ is assigned to the C=O stretching of the amide I band. The band at approximately 870 cm^−1^ is the characteristic band of calcium carbonate. In Figure 6b, the absence of the 870 cm^−1^ band confirms successful demineralization. As shown in Figure 6c, specific chitin bands became more distinct after chitin extraction. A weak C–H stretching vibration from the acetyl group of chitin was observed as a shoulder around 2930 cm^−1^. The characteristic amide I and amide II bands of chitin were detected at approximately 1623 cm^−1^ and 1550 cm^−1^, respectively.

#### 3.4.3. FT-IR Spectra of Chitosan

**Figure 7:**
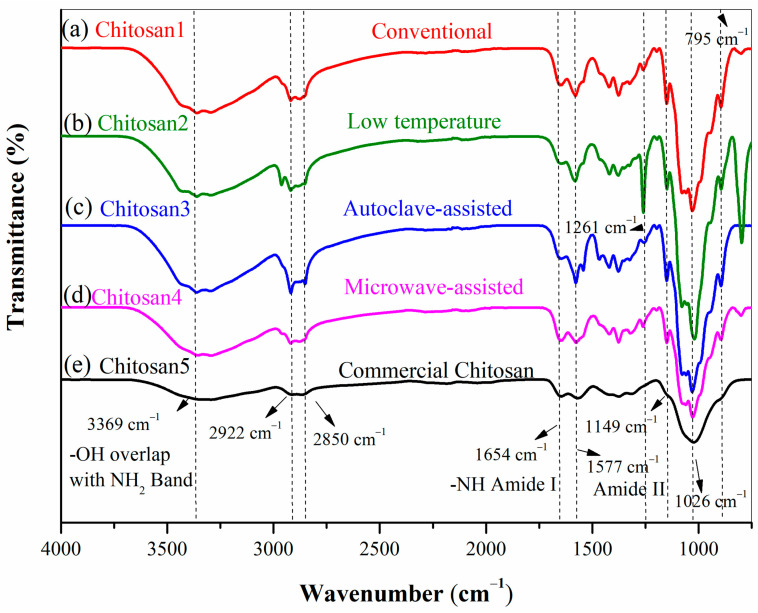
FT-IR spectra of (**a**) chitosan1; (**b**) chitosan2; (**c**) chitosan3; (**d**) chitosan4; and (**e**) chitosan5.

Figure 7 shows the FT-IR spectra of chitosan obtained using the various deacetylation methods. The amide I (1654 cm^−1^) and amide II (1577 cm^−1^) bands are characteristic of chitosan. All spectra exhibit a broad band between 3400 and 3200 cm^−1^. The absorption bands at 2922 and 2850 cm^−1^ correspond to symmetric and asymmetric C–H stretching, respectively. In the conventional method (Figure 7a), only a modest decrease in amide I content was observed. In Figure 7b, notable amide I and II bands were retained, and additional absorptions appeared at 1261 cm^−1^ and 795 cm^−1^. The autoclave-assisted method achieved the most effective deacetylation, which displayed the intense amide II band at ~1577 cm^−1^ and a notably diminished amide I band at 1654 cm^−1^.

**Figure 8:**
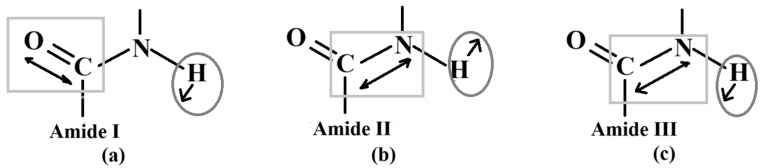
Vibrational modes of (**a**) amide I; (**b**) amide II; (**c**) amide III. Adapted from Ichikawa et al. [44] MDPI, Micro, 2023.

The characteristic vibrational modes of the amide groups in chitosan are shown in Figure 8. The amide I mode (Figure 8a) is primarily attributed to C=O stretching vibrations, whereas the amide II mode (Figure 8b) corresponds to N–H bending coupled with C–N stretching vibrations. The amide III mode (Figure 8c) involves complex C–N stretching and N–H bending vibrations.

#### 3.4.4. Thermal Analysis 

**Figure 9:**
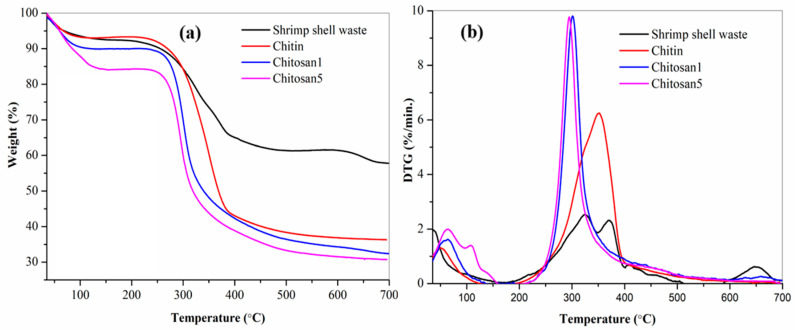
Thermogravimetric analysis (**a**) and differential thermogravimetric analysis (**b**) curve of shrimp shell waste, chitin, chitosan1 and chitosan5.

The thermal behavior of shrimp shell waste, chitin, and chitosan samples was determined by thermogravimetric analysis (TGA) and differential thermogravimetric (DTG) analysis, as shown in Figure 9. All samples exhibited an initial weight loss between 50 and 150 °C, corresponding to the loss of moisture [45,46]. The higher moisture content was exhibited by the chitosan samples (9.9% for Chitosan1 and 15.8% for Chitosan5) compared to chitin (6.4%) and shrimp shell waste (7.4%). SSW demonstrated two distinct decay stages, with maximum degradation temperatures at 323.3 °C and 368.9 °C. Chitin and chitosan samples underwent a single-step decomposition process. Chitin exhibited the highest thermal stability with a maximum decomposition temperature of 351.9 °C. Chitosan1 degraded at 301.3 °C, whereas commercial Chitosan5 decomposed at a slightly lower temperature of 294.9 °C. The residual mass at 700 °C was 57.8% for shrimp shell waste, 37.6% for chitin, 35.9% for Chitosan1, and 31.0% for Chitosan5.

## 4. Discussion

### 4.1. Efficiency of Extraction and Pretreatment Methods

Solar drying was chosen due to its low cost, eco-friendly nature, and ability to reduce unpleasant odors; however, the characteristic pinkish pigmentation of shrimp shell waste largely faded during the process. As an economical and eco-friendly method that can be performed in the open air, solar drying poses no significant challenge to people in rural areas. Various methods, including freeze-drying, superheated steam drying, jet-spouted bed drying, and heat pump drying, are employed to dry shrimp [47]. Particle size reduction significantly enhances solvent penetration and mass transfer, thereby improving extraction yield and reproducibility [28].

Shrimp shells contain substantial amounts of calcium carbonate (CaCO_3_); if inadequately eliminated, the ash content remains high. For commercial applications of chitosan, the ash residue must be below 1% [48,49]. The efficiency of demineralization was evaluated based on the residual ash content.

Table 5 summarizes the process conditions reported in recent years for the extraction of chitin from shrimp shell waste. These studies revealed considerable variations in demineralization, deproteinization, and decolorization methods, including the use of different acids (HCl, citric acid, and acetic acid), varying alkali concentrations, and bleaching agents such as hydrogen peroxide or organic solvents. Chitin yields reported in these studies range widely from approximately 10% to over 39%, reflecting differences in raw materials, processing parameters, and analytical techniques. This overview highlights the lack of standardized protocols in the field and underscores the trade-off between optimizing experimental conditions to maximize yield and ensure product quality.

Solar drying resulted in only superficial bleaching, indicating the limited penetration of the chitin matrix. To the best of our knowledge, no previous study has reported the use of solar energy for chitin decolorization. Although environmentally friendly, this approach is impractical for commercial applications owing to its time-consuming nature and partial effectiveness. Overall, hydrogen peroxide was determined to be the most effective bleaching method among those investigated in this study.

### 4.2. Comparison of Deacetylation Degrees

Conversion of chitin to chitosan involves alkaline deacetylation, which alters the physicochemical and functional characteristics of the polymer [57]. The degree of deacetylation, viscosity, and molecular weight all influence the characteristics of chitosan. Various chemical methods have been developed. The conventional thermochemical method is the most widely used. This method uses 50% NaOH and requires a temperature of at least 100 °C. However, prolonged exposure can lead to a significant decrease in molecular weight; therefore, both the reaction time and NaOH concentration should be optimized to improve process efficiency [18]. In addition to the conventional technique, alternative autoclave and microwave-assisted procedures have been developed. The microwave-assisted method, which operates at lower energy, enables faster completion of the reaction while achieving a high degree of deacetylation. It is based on rapid heating using microwave rays via direct interactions with chitin. As a result, microwave-assisted deacetylation reduces energy consumption, shortens the processing time, and lowers the overall environmental footprint [58]. Adhiksana et al. carried out chitin deacetylation in a 70% NaOH solution using a microwave at 70 °C for 5, 7, 11, and 15 min at power of 350 W. The degree of deacetylation of chitosan increased slightly from 79.5% to 79.96% with an increase in reaction time from 5 to 15 min [59]. Knidri et al. reported in their study that deacetylation required longer times at 90 W and 160 W [21]. However, the conversion of chitin to chitosan began at 8 min and 4 min, using power values of 350 W and 500–650 W, respectively. Deacetylation degrees of 81.3% and 82.8% were obtained by reacting chitin with a 40% NaOH solution for 12 min at 500 W and 650 W, respectively. However, no significant increase was observed when the deacetylation time increased to 14 min. While Knidri et al. reported that 47% and 57% deacetylation degrees were achieved with 14 min deacetylation of chitin extracted from Moroccan shrimp shell waste at 90 W and 160 W [21], in this study, a 67% deacetylation degree was achieved with 15 min deacetylation of chitin extracted from pink shrimp from the Marmara Sea, Türkiye, at 140 W. Sahu et al. reported that when chitin was reacted with a 45% *w*/*v* NaOH solution under 900 W microwave energy, it reached a DD of 85.3% in 5.5 min [42]. Additionally, Ruiz et al. conducted an examination that achieved an 89% deacetylation degree in 3.5 min, with a higher microwave energy of 1800 W [60]. As observed in microwave-assisted deacetylation studies, the degree of deacetylation of chitosan increased with increasing microwave energy and duration, indicating a higher efficiency of autoclave-assisted processes [28].

In this study, the chitosan yield obtained from chitin extracted from Marmara Sea shrimp using the conventional method was determined to be 76%, whereas Tokatlı et al. reported a slightly lower yield of 74%. Furthermore, when 50% NaOH was used for deacetylation, the degree of deacetylation achieved in this study reached 81% at 120 °C within 180 min. In contrast, Tokatlı et al. [28]. reported a comparable degree of deacetylation (80%) at a lower temperature (100 °C), but only after a substantially longer reaction time (720 min). Bhardwaj et al. [61] claimed that an increase in the degree of deacetylation results in a decrease in viscosity. Although Hejazi et al. [62] reported that the viscosity of a chitosan solution increases with the degree of deacetylation; however, no clear correlation between viscosity and degree of deacetylation was found in this investigation. As the degree of deacetylation of chitosan increases, the number of cationic groups in chitosan increases when it dissolves in acidic solutions, leading to greater electrostatic repulsion among the polymer chains. This causes the polymer chains to separate, potentially resulting in reduced viscosity. The degrees of deacetylation of chitosan produced by different methods were compared. The conventional method facilitates large scale production; thus, the thermal, morphological, and biodegradable features of low-molecular-weight chitosan synthesized using this method were examined.

As shown in Table 4, based on the degrees of deacetylation calculated by the titrimetric method, the chitosan sample with a degree of deacetylation of 95% exhibited a solubility of 97.6% in 1% acetic acid, whereas the chitosan with a degree of deacetylation of 64% showed a lower solubility of 77.5%. These results clearly indicate that the solubility of the produced chitosan samples in 1% acetic acid becomes higher with increasing degree of deacetylation. This behavior can be attributed to the higher content of free amino groups at elevated DD values, which undergo protonation in acidic media, thereby enhancing polymer–solvent interactions and facilitating chitosan dissolution [63].

Chitosan solubility is strongly dependent on the pH of the medium. Due to the presence of free amino groups, chitosan becomes protonated under acidic conditions (pH < 6), which enhances solubility through electrostatic repulsion. However, at pH > 6.5, reduced protonation leads to aggregation and precipitation. In addition to pH, solubility is influenced by molecular weight, degree of deacetylation, extraction pretreatments, processing conditions, ionic strength, and acetyl group distribution, allowing chitosan solubility to be tailored for specific applications [64,65,66]. A strong correlation between the degree of deacetylation and solubility has been well-documented in the literature. Hossain and Iqbal (2014) [67] reported that the quality of chitosan chemically extracted from shrimp shell waste is highly dependent on deacetylation conditions. In their study, chitosan obtained using 60% NaOH at 60 °C for 24 h exhibited a degree of deacetylation of 81.2% and a high solubility of 97.6%. In contrast, chitosan deacetylated with 30% and 40% NaOH exhibited lower solubility values, ranging from 48.3% to 71.27%. In contrast, samples treated with 50% and 60% NaOH achieved excellent solubility levels, approaching 97.6% [67].

### 4.3. Interpretation of Characterization Results

Overall, Figure 4 highlights the morphological alterations that occur during the conversion of shrimp shell waste into chitosan. The removal of minerals and proteins results in the synthesis of fibrous chitin; however, deacetylation disrupts this structure and increases surface roughness. Commercial chitosan was more homogeneous than laboratory-prepared chitosan, indicating a higher purity and more controlled processing. These differences in surface characteristics are particularly relevant, as morphology significantly affects the solubility, reactivity, and potential applications of chitosan in biomedical, food, and environmental fields [68,69]. These structural features contributed to a rigid and robust matrix, and distinct grooves resembling vascular channels were observed. The surface appeared rougher and more porous, which is consistent with the structural rearrangement of the polymer chains and localized degradation [17].

The amide I band at 1654 cm^−1^ is attributed to C=O stretching vibrations, and its reduced intensity indicates the removal of acetyl groups after a higher degree of deacetylation [60,70,71]. The absorption band at 1261 cm^−1^ was assigned to O–H bending vibrations in chitosan, while the band at 893 cm^−1^ corresponded to CH bending out of the monosaccharide ring plane [69]. These results indicated that the autoclave approach improved the degree of deacetylation of the synthesized chitosan. Figure 8 highlights the C=O and N–H stretching vibrations within the rectangular regions, while the N–H bending vibrations are indicated by circular markers, in good agreement with literature reports [44].

The increased porosity of the Chitosan1 sample resulting from thermochemical deacetylation is clearly observed in the SEM micrographs in Figure 5c. During the deacetylation process, the cleavage of glycosidic bonds and degradation of acetylated and deacetylated units caused the breakdown of the polymer chains into volatile products, resulting in sharp weight loss in the TGA profiles. The results confirmed the superior thermal stability of Chitosan1 compared to the commercial sample, as shown in Figure 9. The enhanced thermal resistance of Chitosan1 can be attributed to its higher degree of deacetylation, which promotes stronger chain packing and reduces the number of structural defects. In contrast, the lower stability of Chitosan5 was associated with its higher hydrophilicity, likely due to its reduced crystallinity and weaker hydrogen bonding, which facilitates moisture absorption in the amorphous domains. The increased water uptake promotes plasticization of the polymer matrix, reduces chain–chain interactions, and accelerates the thermal scission of glycosidic bonds, thereby lowering the overall thermal stability. Furthermore, the lower degree of deacetylation in Chitosan5 results in a higher proportion of acetyl groups within the structure. Because acetyl groups are relatively hydrophobic, their reduced removal limits the exposure of amino groups that could otherwise form stronger hydrogen bonds, weakening intermolecular interactions, and increasing water accessibility [13,72].

### 4.4. Future Perspectives and Sustainability Challenges in Chitosan Production

Energy-assisted deacetylation methods, including microwave and autoclave-assisted methods, demonstrate the capacity to significantly enhance the efficiency of chitosan production by diminishing reaction duration and total energy consumption. Specifically, autoclave-assisted deacetylation provides a significant level of deacetylation under relatively defined and reproducible conditions, whereas microwave irradiation provides fast volumetric heating that can diminish process durations to only minutes. These characteristics highlight the future potential of both approaches as alternatives to conventional thermochemical conversion in chitosan production. Despite these advantages, several challenges must be addressed before microwave- and autoclave-assisted methods can be widely adopted on an industrial scale. High initial equipment costs, limited reactor capacities, and requirements for highly qualified operational control now prevent their large-scale application. Future efforts should therefore focus on reactor design optimization, continuous-flow processing techniques, and techno-economic assessments to encourage the industrial adoption of these energy-assisted approaches. The need for a high level of alkaline solutions is a typical drawback of both conventional and energy-assisted deacetylation methods when considering the sustainability of the chemical-based chitosan production process. Accordingly, recovering and reusing sodium hydroxide and process water from high-concentration alkaline solutions is critical to improving overall process sustainability. Furthermore, high-concentration alkaline solutions can be employed in agricultural applications such as soil remediation or fertilizer applications after being neutralized in accordance with environmental requirements. Future research on alkaline recovery, reuse, and assessment in other industries following required pretreatment can greatly reduce the environmental impact of chitosan production. As a result, the long-term sustainability of chitosan production will depend not only on the chosen deacetylation technique but also on the integration of circular resource management practices, including alkaline recovery and responsible wastewater management. When all these factors are taken together, the current findings are placed within a broader industrial and sustainability framework and support conclusions regarding the balance between process efficiency, material performance, and practical scalability in low-molecular-weight chitosan production.

## 5. Conclusions

Chitin was successfully extracted from shrimp shell waste and converted into low-molecular-weight chitosan using conventional, microwave-assisted, and autoclave-assisted deacetylation methods. The comparative analysis demonstrated that the autoclave-assisted method was the most effective in terms of deacetylation efficiency, achieving a degree of deacetylation of up to 95%, while microwave-assisted deacetylation enabled rapid chitosan production within 15 min, albeit at a lower degree of deacetylation due to the low applied microwave power. The conventional method provided the most reproducible and scalable route, highlighting its suitability for industrial-scale production. FT-IR analysis confirmed the effective removal of mineral components, supported by the disappearance of calcium carbonate bands and a reduction in ash content to 0.9%. SEM observations revealed a well-defined fibrillar structure in chitin, which became partially disrupted following deacetylation, indicating structural modification during chitosan formation. Thermal analysis further showed that laboratory-synthesized Chitosan1 exhibited higher thermal stability (Tmax = 301.3 °C) compared to commercial chitosan (Tmax = 294.9 °C), demonstrating improved material performance. Overall, this study provides a systematic comparison of deacetylation strategies for producing low-molecular-weight chitosan from shrimp shell waste and clarifies the trade-offs between processing efficiency, material properties, and scalability. The findings confirm that waste-derived chitosan can match or exceed the performance of commercial products, thereby supporting its potential for sustainable and scalable biopolymer production.

## Figures and Tables

**Table 1:** Chitosan sample codes and deacetylation conditions.

Sample Codes	Method	Conditions
Chitosan1	Conventional	120 °C for 3 h.
Chitosan2	Low Temperature	70 °C for 48 h.
Chitosan3	Autoclaved-assisted	121 °C for 1 h.
Chitosan4	Microwave energy-assisted	140 W for 15 min.
Chitosan5	Commercial LMWC	-

**Table 2:** Amounts and percentages of shrimp shell waste (SSW) remain after being treated with 1 M HCl.

Time (h)	Amount of Initial SSW (g)	Amount of Retained SSW After DM (g)	Amount of Retained SSW After DM (%)	Ash Content (%)	Mineral Content of SSW (%)
1	200	99.35	49.7	18.26	54.86
2	200	98.66	49.3	2.42	51.27
4	200	94.65	47.3	1.62	53.06
6	200	90.65	45.3	1.3	54.97
8	200	85.65	42.8	0.90	57.37

**Table 3:** Chemical composition of SSW originating from the Marmara Sea.

Moisture (%)	Mineral (%)	Protein (%)	Chitin (%)	Mineral (%) *		
				Ca	P	Mg
73.2 ± 5.4	54.31 ± 2.29	31.27 ± 2.78	14.42 ± 1.99	6.74	2.16	0.78

* Atomic% values obtained from SEM EDS.

**Table 5:** The conditions for chitin extraction from shrimp shell waste in recent years.

Drying	Demineralization	Deproteinization	Decolorization	Chitin Yield (%)	References
*N.D	7% HCl, 1 h, 40 °C	1 M NaOH, 2 h, 80 °C	10% H_2_O_2_, 50 °C, 30 min.	*N.D	[50]
*N.D	1 M HCl, 18 h, room temp.	1 M NaOH, 18 h, 70 °C	*N.D	39.57	[6]
*N.D	2 M HCl, room temp.	2 M NaOH, 55 °C	*N.D	21.88	[51]
*N.D	0.5 M citric acid, 20 min. Room temp.	2 M NaOH, 48 h, room temp. after ultrasound	30% H_2_O_2_ 3 h Room temp.	10.56	[1]
50 °C overnight in hot air oven	4% HCl, 30 °C, 12 h.	4% NaOH, 45 °C, 24 h.	Acetone/ethanol (1:1)	23	[52]
Air-dried	4% (*w*/*v*) aqueous citric acid, 5 h	boiling water for 3 h	*N.D	13.4	[41]
Room temp.	0.73 M HCl, 132.61 min.	0.95 M NaOH, 60.49 °C, 75.65 min.	35% H_2_O_2_ Overnight at room temp.	10.13	
80 °C, hot air oven, 2–3 d.	2N HCl (1:15), 2 h in an incubator shaker	2 N NaOH (1:20), 2 h 50 °C in an incubator shaker	*N.D	14.72	[53]
Sun 8 h.	2 M NaOH, 1:16 (*w*/*v*)	1 M HCl 1:16 (*w*/*v*)	*N.D	*N.D	[54]
	10% acetic acid 1:40 (*w*/*v*), 4 h, 50 °C.	2% (*w*/*w*) NaOH, 2 h, 80 °C, 1:30 (*w*/*v*).	*N.D	*N.D	[55]
Sun	1 N HCl 30 min. 1:15 (*w*/*v*)	3% NaOH for 15 min at 15 psi/121 °C 1:10 (*w*/*v*).	10% sodium hypochlorite solution for 5 min. 1:10 (*w*/*v*).	*N.D	[56]
Sun	1 N HCl, 1–8 h. 1:15 (*w*/*v*)	1 M NaOH 1–4 h. 1:15 (*w*/*v*)	3% H_2_O_2_	14.42	In this study

*N.D.: not determined.

## Data Availability

The data presented in this study are available upon request from the corresponding author.

## Abbreviations

The following abbreviations are used in this manuscript:

DD	Degree of Deacetylation
DM	Demineralization
DP	Deproteinization
DTG	Differential Thermogravimetric
EDS	Energy-Dispersive Spectroscopy
FT-IR	Fourier-Transform Infrared Spectroscopy
LMWC	Low-Molecular-Weight chitosan
MC	Mineral Content
SEM	Scanning Electron Microscope
SSW	Shrimp Shell Waste
TGA	Thermogravimetric Analysis
UV-Vis	Ultraviolet-Visible spectrophotometry
